# Challenging the Wine Component in Mediterranean Diet Scores: Cognitive Outcomes in Portuguese Adults at High Risk of Dementia

**DOI:** 10.3390/nu17223576

**Published:** 2025-11-15

**Authors:** Andreia Mesquita, Daniela de Sousa, Patrícia Padrão, Ana Rute Costa, Pedro Moreira

**Affiliations:** 1 Faculty of Nutrition and Food Sciences, University of Porto, 4150-180 Porto, Portugal; up202103945@edu.fcna.up.pt (A.M.); patriciapadrao@fcna.up.pt (P.P.); 2 EPIUnit ITR, Institute of Public Health of the University of Porto, University of Porto, Rua das Taipas, n.° 135, 4050-600 Porto, Portugal; daniela.sousa@ispup.up.pt (D.d.S.); arcosta@ispup.up.pt (A.R.C.)

**Keywords:** older adults, diet, Mediterranean, cognition

## Abstract

**Introduction:** The Mediterranean diet (MD) has been linked to better cognition, but evidence in older adults at high dementia risk is limited. Moreover, the traditional Mediterranean Diet Adherence Screener (MEDAS) counts daily wine consumption as a beneficial component, which may distort genuine diet–cognition relationships. **Objective:** Evaluate whether MD adherence, as measured with the original MEDAS (MEDAS-O) versus a version that reverses the wine item (MEDAS-R), is associated with cognitive function in Portuguese adults aged 55–85 years at increased dementia risk. **Methodology:** The sample comprised 75 participants from the NUTRIMIND randomised controlled trial (mean age 70.5 ± 7.0 years). MD adherence was evaluated using the original version of MEDAS (MEDAS-O) and an adapted version with a reverse score in the wine question (MEDAS-R). Cognitive function was assessed via the Montreal Cognitive Assessment (MoCA), Addenbrooke’s Cognitive Examination Revised (ACE-R) and Mini-Mental State Examination (MMSE). Statistical analysis was performed using Analysis of Covariance (ANCOVA) models adjusted for age, sex, BMI, education, and physical activity. **Results:** MEDAS-R was positively associated with better MMSE performance (*p* = 0.043) and showed a borderline association with the MoCA (*p* = 0.051), but not with the ACE-R score (*p* = 0.356). No association was found between MEDAS-O and cognitive function. Better cognitive scores were more frequently observed among participants with higher education (*p* < 0.001). **Conclusions:** Reversing the wine item changes how MEDAS relates to cognitive function. These findings support re-evaluating how wine is scored in MD adherence measures.

## 1. Introduction

The Mediterranean Diet (MD), originally characterised by Ancel Keys in the 1960s, is now recognised as one of the most thoroughly investigated dietary patterns and was inscribed in 2010 on UNESCO’s Intangible Cultural Heritage of Humanity list [1,2,3]. It features a predominantly plant-based nutrient profile, including fruits, vegetables, whole grains, tubers, legumes, and nuts, with extra-virgin olive oil as the principal source of fat. Dairy, white meats, fish, and eggs are consumed in moderation, while red and processed meats, as well as sweets, are eaten in limited quantities. Wine, when consumed, traditionally accompanies meals in low–to–moderate amounts [2,3]. Convivial, seasonal, and minimally processed cooking methods further characterise this eating pattern [3].

Beyond its cultural and nutritional significance, the MD has gained increased attention for its potential role in promoting healthy ageing and preserving cognitive function. Currently, approximately 25% of Europeans are aged 60 years or older (the highest global proportion), which is projected to double by 2050 [4]. In Portugal, the ageing index reached 188 older adults per 100 young people in 2023 [5].

Ageing is associated with significant health challenges, among which cognitive decline is one of the most concerning [6]. A study of Portuguese adults aged 65–85 years estimated an incidence of 27 new cases of cognitive impairment per 1000 person-years [7], with downstream increases in dementia, disability, hospitalisation and mortality [6,8].

Robust epidemiological evidence links greater MD adherence to reduced risk of chronic disease and to preserved cognitive function; specifically, improvements in global cognition, gait speed, lower limb strength, and memory have been reported among older adults without dementia [9,10,11,12]. However, most studies target non-Portuguese cohorts and often rely on a single cognitive instrument, which limits external validity and the depth of cognitive characterisation in our population. Moreover, whereas the cross-national, validated 14-item Mediterranean Diet Adherence Screener (MEDAS) [13] awards positive points for daily wine consumption, another national validation of the same questionnaire, conducted by telephone, reverses this scoring [14]. These inconsistencies in how wine consumption is scored reflect the ongoing debate regarding its role in cardiovascular and cognitive health. While moderate wine consumption, traditionally considered a component of the MD, has been associated with potential cardiovascular and neuroprotective benefits [15,16], more recent research has highlighted possible neurotoxic effects of alcohol even at low amounts [17,18].

To address these inconsistencies and expand the current evidence in a Portuguese context, the present study focused on older adults at increased risk of dementia. Participants were considered to be at increased risk of dementia according to their Cardiovascular Risk Factors, Ageing, and Dementia (CAIDE) risk score, which incorporates the variables age, sex, education, systolic blood pressure, body mass index, total cholesterol and physical activity [19].

In this context, this study aims to examine the association between adherence to the MD, with daily wine consumption scored positively vs. reverse-scored, and cognitive function in Portuguese older adults, using three complementary assessment tools: the Montreal Cognitive Assessment (MoCA), the Addenbrooke’s Cognitive Examination Revised (ACE-R) and the Mini-Mental State Examination (MMSE).

## 2. Materials and Methods

### 2.1. Study Design and Participants

This cross-sectional analysis was conducted using preliminary baseline data collected between January and May 2025 from the NUTRIMIND project (ClinicalTrials.gov NCT06853405) and included a sample of 75 older adult participants from Porto, Portugal. Participants were recruited either through referrals by healthcare professionals from primary care units or through self-enrolment following publicity via community channels.

To be enrolled in the project, individuals had to meet the following criteria: be aged between 55 and 85 years; have a higher risk of developing dementia based on their CAIDE risk score (score ≥ 6 points) [19]; and have an educational attainment of at least four years. Exclusion criteria included a MoCA [20] score lower than the validated cutoff points (defined as two standard deviations (SD) below the normative reference value for the corresponding age and education in the Portuguese population); any medical condition that could limit the participation in the intervention (e.g., blindness, amputation…); lack of autonomy in performing daily activities or a confirmed diagnosis of dementia or major disability.

Ethical approval for the study was granted by the Ethics Committee of the Northern Region Health Administration (Approval Number: CE/2023/114) and by the Data Protection Officer of the *Instituto de Saúde Pública da Universidade do Porto*.

### 2.2. Assessment of Cognitive Function

Cognitive function was assessed using three instruments, administered by a trained interviewer: the MoCA [20], the ACE-R [21,22] and the MMSE [23].

The MoCA is a preferred tool for detecting cognitive decline in its early stages. It has been culturally and linguistically adapted for the Portuguese population and validated in older adults. The MoCA evaluates eight cognitive domains (Visuospatial/Executive, Naming, Memory, Attention, Language, Abstraction, Delayed Recall and Orientation). Higher total scores (maximum: 30 points) are indicative of better cognitive functioning. A score that falls more than 1.5 SD below the normative mean for age and education is considered suggestive of Probable Cognitive Impairment (PCI) [20].

The ACE-R has also been adapted and validated for Portuguese adults [22]. This tool assesses five domains of neurocognitive functioning (Attention and Orientation, Memory, Verbal Fluency, Language, and Visual-Spatial Ability), and the final score ranges from 0 to 100 points. The total score is the sum of the domain scores, with higher scores reflecting better cognitive function. As with the MoCA, a score more than 1.5 SD below the normative mean for age and education was considered suggestive of PCI [21]. The ACE-R incorporates items from another well-known cognitive screening tool—the MMSE [23]. The MMSE consists of six sections that assess various cognitive domains, with a maximum total score of 30 points. As with the previous tools, PCI was identified by an MMSE score more than 1.5 SD below the age- and education-adjusted normative mean [23].

### 2.3. Adherence to the MD

Adherence to the MD was assessed using the MEDAS, developed within the framework of the PREDIMED study [24]. This instrument consists of 14 questions related to dietary habits, each scored either 0 or 1, with a maximum total score of 14 points. A score of 10 or above was considered indicative of high adherence to the MD. The MEDAS was initially validated for the Spanish population (aged 55–80 years) [24] and was later adapted for use in various populations [13], including Portuguese adults, in both in-person and telephone formats; this Portuguese version demonstrated moderate reliability and validity [14]. In the present study, we used the original scoring scheme, in which 1 point is assigned for moderate wine consumption (7 to 14 glasses of 100 mL/week) [13,20], and for clarity, we will refer to this version as MEDAS-O (Original). The key alternative version, validated for telephone use in Portugal, applies reverse scoring for wine, assigning 1 point when the frequency of wine consumption is <1 portion per day [14]; we will refer to this as MEDAS-R (Reverse). To account for the wine component, we calculated adherence scores using both MEDAS-O and MEDAS-R scoring schemes to assess the impact of the wine component on the associations with cognitive outcomes.

### 2.4. Sociodemographic and Lifestyle Variables

Sociodemographic and lifestyle information was collected via structured questionnaires. Age was recorded in years and grouped into three categories: <65, 65–74, and ≥75 years. Measured Body Mass Index (BMI) was calculated from weight and height and classified according to World Health Organisation (WHO) criteria [25]: underweight (<18.5 kg/m^2^), normal weight (18.5–24.9 kg/m^2^), overweight (25.0–29.9 kg/m^2^), and obesity (≥30.0 kg/m^2^). Educational attainment was reported in completed years of schooling and categorised as ≤4, 5–9, and ≥10 years. Monthly income was self-reported and defined as ≤EUR 1000, EUR 1001–1500, EUR 1501–2000, and >EUR 2000. Smoking status was classified as current smoker, former smoker, or never-smoker. Physical activity level was determined from self-reported frequency of structured exercise, with “active” participants defined as those engaging in at least 20 min of exercise that induced sweating and breathlessness on two or more occasions per week [19].

### 2.5. Statistical Analysis

Continuous variables were assessed for normality by visual inspection of histograms and Q–Q plots. As none of the cognitive function measures were normally distributed, results are reported as medians and 25–75th percentiles (P25–P75). Categorical variables were expressed as absolute and relative frequencies (n, %). Group differences in sociodemographic, lifestyle, and health-related characteristics according to adherence to the MD and cognitive function (both treated as a binary variable) were evaluated using the chi-square (χ^2^) or Fisher’s exact tests, as applicable. The comparison of the same participants’ characteristics according to cognitive function (treated as a continuous score) was examined by the Mann–Whitney or Kruskal–Wallis tests, as appropriate.

Associations between adherence to the MD (both MEDAS-O and MEDAS-R, treated as continuous independent variables) and the scores of each cognitive assessment tool (continuous dependent variables) were examined using Analysis of Covariance (ANCOVA) models adjusted for age, sex, BMI, education, and physical activity level, selected a priori according to prior literature. Before this analysis, and due to their non-normal distribution, continuous cognitive outcomes were all log-transformed. Statistical significance was set at *p* < 0.05 (two-tailed). Additionally, exploratory analyses examining potential interactions between sex and both MEDAS-O and MEDAS-R scores were conducted and presented in the Supplementary Materials (Table S1). All analyses were performed using IBM SPSS Statistics, version 29.0.2.0 (IBM Corp., Armonk, NY, USA).

## 3. Results

This analysis included 75 participants, 84.0% of whom were female, with a mean age of 70.5 years (SD = 7.0) and 50.7% aged 65–74 years; 33.0% of participants were classified as overweight, and 21.0% as obese. Educational attainment was 4 years in 13.3%, 5–9 years in 17.3%, and ≥10 years in 69.3% of the sample, and the monthly income exceeded EUR 2000 in 56.7%. It was also found that only 4% were current smokers, and just 17% engaged in structured moderate-to-vigorous physical activity at least twice a week. Regarding MEDAS-O, 17.3% of participants exhibited high adherence. However, when the wine scoring was reversed, high adherence increased to 33.3%. Nonetheless, MEDAS scores showed no significant associations with any sociodemographic or lifestyle variable—sex, age, BMI, education level, monthly income, smoking status or physical activity level. These results are presented in Table 1.

PCI was observed in 9.3% on both the MoCA and ACE-R and in 54.7% on the MMSE. Based on bivariate analyses, education was more frequently observed among cognitive scores across all measures (MoCA *p* < 0.001; ACE-R *p* < 0.001; MMSE *p* = 0.001). Monthly income (MoCA *p* = 0.011; ACE-R *p* = 0.006; MMSE *p* = 0.011) and smoking status (MoCA *p* = 0.023; ACE-R *p* = 0.008; MMSE *p* = 0.03) were also significantly associated with cognitive function, as demonstrated in Table 2. Age showed a significant association only with the ACE-R scores (*p* = 0.013). Whereas sex, BMI, and physical activity did not reach statistical significance in relation to any of the cognitive measures.

The ANCOVA models, adjusted for sex, age, education, BMI and physical activity level (Table 3), revealed no significant association between total MEDAS-O score and any cognitive measure. Specifically, MEDAS-O adherence was not related to MoCA performance (β = 0.006; 95% CI [−0.001 to 0.014]; *p* = 0.107), ACE-R total (β = 0.003; 95% CI [−0.003 to 0.009]; *p* = 0.283) or MMSE score (β = 0.005; 95% CI [−0.001 to 0.011]; *p* = 0.087). When examining the association with MEDAS-R, the MoCA showed a trend that did not reach statistical significance (β = 0.008; 95% CI [−0.00005 to 0.015]; *p* = 0.051). Similarly, the ACE-R estimate did not reach statistical significance (β = 0.003; 95% CI [−0.003 to 0.008]; *p* = 0.356). In contrast, MEDAS-R demonstrated a statistically significant positive association with the MMSE (β = 0.006; 95% CI [0.000 to 0.012]; *p* = 0.043).

Of the covariates examined, only years of education was significantly associated with cognitive outcomes (*p* < 0.001), accounting for 18.6–35.5% of variance. Sex, age, BMI and physical activity level were non-significant contributors.

## 4. Discussion

The present study found no significant association between MEDAS-O score and the MoCA, ACE-R, or MMSE scores, which contrasts with earlier research linking higher MD adherence with better cognition [26,27]. However, when applying the MEDAS with reversed wine scoring (MEDAS-R), a statistically significant association with MMSE scores was observed, and a borderline association with MoCA was also observed. This finding highlights the need for further studies with larger samples to clarify the potential cognitive relevance of this dietary pattern.

To the best of our knowledge, this is the first study to examine the relationship between adherence to the MD, as assessed using both the MEDAS-O and the MEDAS-R, and cognitive function in older adults. The significant findings observed with the MEDAS-R, in contrast to the non-significant results with MEDAS-O, suggest that the scoring of wine consumption may meaningfully influence observed Mediterranean diet–cognition relationships in this population, which supports a cautious approach to alcohol in MD scoring instruments.

The broader effect of alcohol on health has been extensively debated. A previous analysis of 21,000 participants aged 40–70 years from the United Kingdom demonstrated that even moderate alcohol intake (more than 50 g of ethanol per week) correlates with increased brain iron deposition and that elevated iron levels predict poorer executive function and slower information processing speed [28]. Similarly, a longitudinal cohort study in China, involving 5354 older adults, reported that drinkers faced a 29% greater risk of cognitive decline vs. abstainers, with risk proportional to alcohol dose consumption [29]. However, there is evidence that contradicts these discoveries. In a prospective cohort of 19887 adults from the United States, low-to-moderate alcohol consumption [defined as fewer than eight standard drinks per week for women and fewer than 15 for men (14 g of ethanol per drink)] was associated with preserved cognitive function as measured by the total cognitive score and by domain-specific scores in mental status, word recall and vocabulary. Moreover, compared with abstainers, low-to-moderate drinkers exhibited significantly slower rates of cognitive decline over time across all evaluated domains [30], and low-volume drinking (defined as 1.30–24.99 g/d of alcohol intake) is associated with the lowest risk of coronary heart disease in individuals over 55 years of age [31]. Nevertheless, consistent with the Global Burden of Disease Study 2021, alcohol consumption was responsible for approximately 2.4 million deaths in 2020, with nearly half of all alcohol-attributable cancer deaths in the European Region occurring among individuals who consumed up to one standard drink per day [32]. In addition, according to the WHO, alcohol is classified as a carcinogen and is associated with various types of cancer, liver diseases, increased cardiovascular risk and mental health problems such as depression and suicide [18].

This controversy in the literature raises the question of whether moderate wine consumption should continue to be promoted as an integral component of the MD, given its potential deleterious effects on health when considered from a holistic perspective. Although some earlier studies linked moderate wine intake with lower cardiovascular risk [33], the latest evidence suggests no cardiovascular benefit from moderate alcohol consumption and possible harm even at low doses [34]. Furthermore, the Global Burden of Disease analyses have challenged any health advantage of alcohol, and alcohol is classified as a group I carcinogen with no clear threshold for certain cancers [32,35]. This evidence underpins the World Health Organisation’s statement that there is no safe level of alcohol consumption for health [36]. In line with this position, we prioritised an adapted MEDAS that does not award a positive point for daily wine intake. We refrain from recommending initiation or escalation of alcohol use in any population group. Among current older drinkers, any potential ‘protective signal’ should be viewed strictly as harm reduction within a traditional pattern (with meals, without binge drinking), rather than as proof of health benefit. We acknowledge that a definitive resolution of this controversy will require large, randomised evidence, such as the ongoing UNATI trial, and until then, our stance remains cautious.

It is worth noting that our results, which support the association between MEDAS-R and cognitive function, align with previous research. A recent meta-analysis reported that greater adherence to the MD reduced the rate of cognitive decline by approximately 18% and that participants following the MD maintained better cognitive function over time. These findings provide robust evidence supporting the protective role of the MD in lowering the risk of cognitive decline, dementia, and Alzheimer’s disease [26]. Additionally, a 2010 systematic review concluded that high MD adherence is associated with attenuated cognitive decline, lower conversion rates from mild cognitive impairment to Alzheimer’s disease, and reduced Alzheimer’s disease incidence [37,38].

In addition, our study revealed a marked discrepancy emerged between sociodemographic status, lifestyle behaviours and MD adherence: although approximately 70% had a high educational attainment (≥10 years) and 57% reported a monthly income exceeding EUR 2000, overall, adherence to the MD was unexpectedly low, with only 17% meeting criteria for high MD adherence under the MEDAS-O scoring. This contrasts with numerous studies demonstrating that higher socioeconomic status, education and physical activity predict greater MD adherence in older populations [39].

However, low adherence to the MD among older adults has already been documented in the literature. For instance, a sample of 609 Portuguese older adults in the Nutrition UP 65 cross-sectional study found that only 43% of participants adhered to the MD [40]. Likewise, the European Health Interview Survey identified a weak but statistically significant negative correlation between age and MD adherence, thereby reinforcing the decrease in adherence with advancing age [41]. After applying the MEDAS-R, the proportion of participants classified as high adherents increased from 17% to 33%. This rise may be attributable to the predominance of female participants in our sample, as, according to the National Food, Nutrition, and Physical Activity Survey of the Portuguese General Population 2015–2016 [42], elderly Portuguese women consume, on average, only 37 g of wine per day, compared with 270 g among their male counterparts. Moreover, the potential influence of sex differences deserves further attention. In our sample, women reported substantially lower wine intake than men, which may have contributed to the differences observed between MEDAS-O and MEDAS-R classifications. Additionally, exploratory analyses examining potential interactions between sex and Mediterranean diet adherence revealed no significant interactions for any cognitive outcome when using the MEDAS-O (all *p* > 0.05). However, when applying the MEDAS-R, marginally stronger interactions emerged for ACE-R (*p* = 0.071) and MMSE (*p* = 0.074), suggesting a possible sex-specific pattern in the association between MEDAS-R and cognitive performance. The alcohol component effectively serves as a barrier, penalising individuals who otherwise adhere to MD patterns but abstain from or restrict alcohol consumption.

These findings may have implications for both research and clinical practice. Specifically, they suggest that current MD adherence tools, such as MEDAS, could benefit from revision, particularly regarding the treatment of alcohol consumption.

Regarding cognitive function, PCI was observed in 9.3% on the MoCA and ACE-R and in 54.7% on the MMSE. Both the MoCA and ACE-R demonstrate broad construct validity by encompassing executive, attentional, and visuospatial domains. The ACE-R is even more comprehensive than the MoCA, assessing additional cognitive areas and thus being less susceptible to false positives, whereas the MMSE focuses on orientation and immediate recall, which may predispose it to such errors. Moreover, MMSE orientation items are highly sensitive to mental disorders such as anxiety and depression, and it has been demonstrated that older adults with elevated symptoms of these conditions exhibit significantly reduced orientation subscale scores [43]. Finally, the predominance of women in our sample and the higher prevalence of anxiety and depressive disorders among females [44] may overestimate MMSE PCI detection relative to MoCA/ACE-R assessments.

The combined use of the MoCA, ACE-R, and MMSE allowed for a broader and more robust assessment of global cognition than would be possible with a single tool. Although each of these instruments serves primarily as a screening measure, they differ slightly in sensitivity and cognitive emphasis, capturing complementary aspects of cognitive performance. This multidimensional approach reduces potential measurement bias, enhances construct validity, and improves the likelihood of detecting subtle cognitive differences among older adults at increased risk of dementia.

As regards education, it accounted for the largest proportion of variance in cognitive function in the multivariate models, followed by monthly income and smoking status. These findings are corroborated by studies showing that greater years of formal education predict superior late-life cognitive performance [10], higher income is associated with better cognitive outcomes in adults aged 65 years or older [45], and current smoking confers a significantly increased risk of dementia and accelerates cognitive decline compared to former or never smokers [46,47]. Although physical activity had no significant effect on any cognitive measure in our sample, existing literature demonstrates that structured exercise induces measurable improvements in cognition and mental health [48].

Finally, it should be acknowledged that the cognitive tests applied (MMSE, MoCA, and ACE-R) are screening tools rather than comprehensive neuropsychological batteries, which may have limited the detection of subtle domain-specific differences.

Lastly, several limitations must be acknowledged. Given the modest sample size and the number of covariates included in the adjusted models, statistical power may be limited. Therefore, the findings should be interpreted with caution. The dichotomisation of variables in our study also warrants careful consideration. Although dichotomising physical activity enhances model parsimony and reduces overfitting risk while maintaining interpretability, it also reduces sensitivity to detect dose–response relationships. Nevertheless, physical activity was defined using a previously established and reproducible criterion, providing a pragmatic indicator of regular exercise in epidemiological research. Thus, we believe that the main associations observed are unlikely to be substantially affected by this simplification. Similarly, dichotomising cognitive scores using established clinical cutoffs for PCI facilitated interpretation and comparison with prior research but may have introduced misclassification for individuals near threshold values and reduced sensitivity to detect subtle cognitive variations. However, the consistency of results across the three cognitive measures supports the robustness of the observed associations. Furthermore, our sample was predominantly self-selected via media channels and senior universities, with inclusion criteria requiring at least four years of education and excluding individuals with severe baseline cognitive impairment. These decisions introduce potential selection bias, restrict score variability and limit the representativeness of the Portuguese population. Coupled with a modest sample size and a cross-sectional design, causal or longitudinal inferences were precluded, possibly obscuring the subtle benefits of MD. Nevertheless, thorough covariate adjustment supports these cross-sectional findings. Additionally, data on medication use that may affect cognition were not available, which may have introduced residual confounding. Future studies should capture and adjust for these medications. Moreover, future analyses should explore the relationship between alcohol consumption and cognitive function, considering continuous scales to capture better dose effects and potential differences by sex, age, nutritional and health status. A simple yes/no item may lack sensitivity in distinguishing consumption patterns and the sociocultural contexts in which alcohol is consumed, a key element of Mediterranean conviviality.

## 5. Conclusions

In older Portuguese adults at increased dementia risk, reversing the wine component of the MEDAS revealed a positive association between MD adherence and cognitive performance. These preliminary findings underscore the importance of reconsidering the inclusion of small to moderate wine consumption (one or two glasses per day) as a beneficial component in MD adherence scores. Given the evolving evidence on alcohol-related health risks, future research with larger, more diverse samples is warranted to confirm these results and inform the refinement of dietary assessment tools used in cognitive ageing research.

## Figures and Tables

**Table 1:** Sociodemographic and lifestyle characteristics by Mediterranean Diet adherence.

		Adherence to MEDAS-O			Adherence to MEDAS-R		
All Participants	n (%)	Low Adherence n (%)	High Adherence n (%)	*p*-Value	Low Adherence n (%)	High Adherence n (%)	*p*-Value
(n = 75)		62 (82.7)	13 (17.3)		50 (66.7)	25 (33.3)	
**Sex**				0.679			0.740
Female	63 (84.0)	51 (82.3)	12 (92.3)		41 (82.0)	22 (88.0)	
Male	12 (16.0)	11 (17.7)	1 (7.7)		9 (18.0)	3 (12.0)	
**Age** (years)				0.438			0.686
<65	14 (18.7)	11 (17.7)	3 (23.1)		9 (18.0)	5 (20.0)	
65–74	38 (50.7)	30 (48.4)	8 (61.5)		24 (48.0)	14 (56.0)	
≥75	23 (30.7)	21 (33.9)	2 (15.4)		17 (34.0)	6 (24.0)	
**BMI** (kg/m^2^)				0.439			0.871
Underweight	2 (2.7)	1 (1.6)	1 (7.7)		1 (2.0)	1 (4.0)	
Normal weight	32 (42.7)	25 (40.3)	7 (53.8)		21 (42.0)	11 (44.0)	
Overweight	25 (33.3)	22 (35.5)	3 (23.1)		18 (36.0)	7 (28.0)	
Obesity	16 (21.3)	14 (22.6)	2 (15.4)		10 (20.0)	6 (24.0)	
**Education Level, years**				1			0.121
4	10 (13.3)	8 (12.9)	2 (15.4)		9 (18.0)	1 (4.0)	
5–9	13 (17.3)	11 (17.7)	2 (15.4)		10 (20.0)	3 (12.0)	
≥10	52 (69.3)	43 (69.4)	9 (69.2)		31 (62.0)	21 (84.0)	
**Monthly income** (EUR)				0.11			0.354
≤1000	11 (18.3)	10 (21.3)	1 (7.7)		9 (25.0)	2 (8.3)	
1001–1500	12 (20)	11 (23.4)	1 (7.7)		7 (19.4)	5 (20.8)	
1501–2000	3 (5.0)	0(0.0)	3 (23.1)		1 (2.8)	2 (8.3)	
>2000	34 (56.7)	26 (55.3)	8 (61.5)		19 (52.8)	15 (62.5)	
**Smoking status**				0.138			0.519
Smoker	3 (4.0)	1 (1.6)	2 (15.4)		1 (2.0)	2 (8.0)	
Former smoker	25 (33.3)	21 (33.9)	4 (30.8)		17 (34.0)	8 (32.0)	
Never smoker	47 (62.7)	40 (64.5)	7 (53.8)		32 (64.0)	15 (60.0)	
**Physical activity level**				0.687			0.666
≥2 times per week	13 (17.3)	10 (16.1)	3 (23.1)		8 (16.0)	5 (20.0)	
<2 times per week	62 (82.7)	52 (83.9)	10 (76.9)		42 (84.0)	20 (80.0)	

BMI—Body Mass Index; MEDAS-O—Mediterranean Diet Adherence Screener-Original; MEDAS-R—Mediterranean Diet Adherence Screener-Reverse scoring in wine. Note: Percentages are calculated within columns and therefore sum to 100% in each column.

**Table 2:** Sociodemographic and lifestyle characteristics by cognitive function assessed by MoCA, ACE-R and MMSE.

	Cognitive Function Assessed by MoCA			MoCA Score		Cognitive Function Assessed by ACE-R			ACE-R Score		Cognitive Function Assessed by MMSE			MMSE Score	
All Participants	Normal Cognition n (%)	PCI n (%)	*p*-Value	Median P25–P75	*p*-Value	Normal Cognition n (%)	PCI n (%)	*p*-Value	Median P25–P75	*p*-Value	Normal Cognition n (%)	PCI n (%)	*p*-Value	Median P25–P75	*p*-Value
(n = 75)	68 (90.7)	7 (9.3)		26.0 24.0–27.0		68 (90.7)	7 (9.3)		91 85–94		34 (45.3)	41 (54.7)		27 25.0–29.0	
**Sex**			1		0.251			0.310		0.111			0.205		0.218
Female	57 (83.8)	6 (85.7)		25.0 22.0–27.0		58 (85.3)	5 (71.4)		91.0 85.0–94.0		31 (91.2)	32 (78.0)		27.0 26.0–29.0	
Male	11 (16.2)	1 (14.3)		23.5 20.3–25.8		10 (14.7)	2 (28.6)		89.5 77.3–91.8		3 (8.8)	9 (22.0)		27.0 25.0–27.8	
**Age** (years)			0.763		0.056			0.319		**0.013**			0.863		0.508
<65	12 (17.6)	2 (28.6)		25.5 20.8–27.5		13 (19.1)	1 (14.3)		91.0 86.0–94.5		7 (20.6)	7 (17.1)		27.5 25.75–29.0	
65—74	35 (51.5)	3 (42.9)		25.5 22.8–27.0		36 (52.9)	2 (28.6)		91.5 89.0–95.0		16 (47.1)	22 (53.7)		27.0 26.0–29.0	
≥75	21 (30.9)	2 (28.6)		23.0 18.0–25.0		19 (27.9)	4 (57.1)		85.0 72.0–92.0		11 (32.4)	12 (29.3)		27.0 25.0–28.0	
**BMI** (kg/m^2^)			0.436		0.911			0.109		0.295			0.776		0.892
Underweight	2 (2.9)	0 (0.0)		23.0 20.0–.		2 (2.9)	0(0.0)		87.0 84.00–.		1 (2.9)	1 (2.4)		26.5 26.0–.	
Normal weight	29 (42.6)	3 (42.9)		25.0 21.3–27.0		30 (44.1)	2 (28.6)		93.0 85.0–94.8		14 (42.2)	18 (43.9)		27.0 26.0–29.0	
Overweight	24 (35.3)	1 (14.3)		25.0 22.0–26.0		24 (35.3)	1 (14.3)		90.0 83.5–92.5		10 (29.4)	15 (36.6)		27.0 25.0–29.0	
Obesity	13 (19.1)	3 (42.9)		25.5 19.0–27.0		12 (17.6)	4 (57.1)		90.0 70.3–94.5		9 (26.5)	7 (17.1)		28.0 23.3–29.8	
**Education Level** (years)			**0.013**		**<0.001**			**0.027**		**<0.001**			0.098		**0.001**
≤4	9 (13.2)	1 (14.3)		19.5 15.8–21.3		10 (14.7)	0 (0.0)		80.5 67.5–85.3		3 (8.8)	7 (17.1)		25.0 21.8–27.5	
5–9	9 (13.2)	4 (57.1)		22.0 18.0–25.5		9 (13.2)	4 (57.1)		84.0 69.0–92.5		3 (8.8)	10 (24.4)		26.0 23.0–27.5	
≥10	50 (73.5)	2 (28.6)		26.0 24.0–27.0		49 (72.1)	3 (42.9)		92.0 90.0–95.0		28 (82.4)	24 (58.5)		28.0 26.0–29.0	
**Monthly income** (EUR)			0.006		**0.011**			0.317		**0.006**			0.128		**0.011**
≤1000	11 (19.6)	0(0.0)		26.0 23.0–27.0		11 (19.3)	0(0.0)		93.0 90.0–95.0		4 (12.9)	7 (24.1)		28.0 26.0–29.0	
1001–1500	9 (16.1)	3 (75.0)		22.5 20.5–24.8		10 (17.5)	2 (66.7)		89.0 83.0–91.0		4 (12.9)	8 (27.6)		25.5 24.0–27.0	
1501–2000	2 (3.6)	1 (25.0)		21.0 14.0–.		3 (5.3)	0(0.0)		88.0 80.0–.		1 (3.2)	2 (6.9)		23.0 20.0–26.0	
>2000	34 (60.7)	0(0.0)		26.0 24.8–27.0		33 (57.9)	1 (33.3)		92.0 91.0–94.0		22 (71.0)	12 (41.4)		27.5 26.0–29.0	
**Smoking status**			0.561		**0.023**			0.561		**0.008**			**0.033**		**0.03**
Smoker	3 (4.4)	0 (0.0)		29.0 27.0–.		3 (4.4)	0 (0.0)		96.0 95.0–.		3 (8.8)	0 (0.0)		30.0 29.0–.	
Former smoker	24 (35.3)	1 (14.3)		25.0 22.0–27.0		24 (35.3)	1 (14.3)		92.0 88.5–95.0		14 (41.2)	11 (26.8)		28.0 26.0–29.0	
Never smoker	41 (60.3)	6 (85.7)		24.0 20.0–26.0		41 (60.3)	6 (85.7)		90.0 83.0–93.0		17 (50.0)	30 (73.2)		27.0 25.0–28.0	
**Physical activity level**			0.597		0.768			1		0.388			0.197		0.358
≥2 times per week	11 (16.2)	2 (28.6)		25.0 20.5–27.5		12 (17.6)	1 (14.3)		91.0 85.5–95.0		8 (23.5)	5 (12.2)		28.0 25.0–30.0	
<2 times per week	57 (83.8)	5 (71.4)		25.0 22.0–27.0		56 (82.4)	6 (85.7)		90.0 84.0–93.0		26 (76.5)	36 (87.8)		27.0 25.75–29.0	

BMI—Body Mass Index; MoCA—Montreal Cognitive Assessment; ACE-R—Addenbrooke’s Cognitive Examination Revised; MMSE—Mini-Mental State Examination; PCI—Probable Cognitive Impairment. Note: Percentages are calculated within columns and therefore sum to 100% in each column; “.” denotes that the percentile was not calculated due to insufficient data. Statistically significant results are shown in **bold** (*p* < 0.05).

**Table 3:** Associations between Mediterranean Diet adherence and cognitive function assessed by Moca, ACE-R and MMSE.

		**MoCA Score**			**ACE-R Score**			**MMSE Score**		
		***p-*** **Value**	**η^2^*p***	**β** **(95% CI)**	***p-*** **Value**	**η^2^*p***	**β** **(95% CI)**	***p-*** **Value**	**η^2^*p***	**β** **(95% CI)**
MEDAS-O		0.006 (−0.001 to 0.014)	0.107	0.039	0.003 (−0.003 to 0.009)	0.283	0.017	0.005 (−0.001 to 0.011)	0.087	0.044
Sex (ref: males)		−0.011 (−0.049 to 0.027)	0.569	0.005	0.003 (−0.024 to 0.030)	0.845	0.001	0.007 (−0.022 to 0.035)	0.653	0.003
Age		−0.001 (−0.003 to 0.001)	0.339	0.011	−0.001 (−0.003 to 0.000)	0.134	0.034	0.000 (−0.002 to 0.001)	0.665	0.003
BMI		0.001 (−0.004 to 0.002)	0.633	0.003	−0.002 (−0.004 to 0.000)	0.074	0.047	−0.002 (−0.004 to 0.001)	0.126	0.035
Education (ref: ≥10 years)	≤4	−0.113 (−0.154 to −0.073)	**<0.001**	0.319	−0.064 (−0.093 to −0.035)	**<0.001**	0.227	−0.051 (−0.082 to −0.020)	**0.002**	0.142
	5−9	−0.057 (−0.094 to −0.021)	**0.003**	0.129	−0.045 (−0.071 to −0.018)	**0.001**	0.149	−0.036 (−0.064 to −0.008)	**0.013**	0.089
Physical activity level (ref: <2 times per week)		−0.004 (−0.41 to 0.032)	0.811	0.001	0.005 (−0.021 to 0.031)	0.702	0.002	0.010 (−0.017 to 0.038)	0.456	0.008
**Model summary**		R^2^ = 0.430	Adj. R^2^ = 0.370		R^2^ = 0.416	Adj. R^2^ = 0.354		R^2^ = 0.303	Adj. R^2^ = 0.229	
		**MoCA Score**			**ACE-R Score**			**MMSE Score**		
		**β** **(95% CI)**	***p-*** **Value**	**η^2^*p***	**β** **(95% CI)**	***p-*** **Value**	**η^2^*p***	**β** **(95% CI)**	***p-*** **Value**	**η^2^*p***
MEDAS-R		0.008 (−0.00005 to 0.015)	0.051	0.056	0.003 (−0.003 to 0.008)	0.356	0.013	0.006 (0.000 to 0.012)	**0.043**	0.060
Sex (ref: males)		−0.012 (−0.050 to 0.025)	0.523	0.006	0.002 (−0.025 to 0.029)	0.863	0.000	0.006 (−0.023 to 0.034)	0.700	0.002
Age		−0.001 (−0.003 to 0.001)	0.385	0.011	−0.001 (−0.003 to 0.000)	0.107	0.039	0.000 (−0.002 to 0.001)	0.645	0.003
BMI		−0.001 (−0.004 to 0.002)	0.649	0.003	−0.002 (−0.004 to 0.000)	0.076	0.047	−0.002 (−0.004 to 0.001)	0.129	0.035
Education (ref: ≥10 years)	≤4	−0.106 (−0.147 to −0.065)	**<0.001**	0.286	−0.062 (−0.092 to −0.032)	**<0.001**	0.207	−0.046 (−0.077 to −0.014)	**0.005**	0.113
	5−9	−0.054 (−0.090 to −0.017)	**0.005**	0.113	−0.044 (−0.070 to −0.017)	**0.002**	0.140	−0.033 (−0.061 to −0.005)	**0.023**	0.075
Physical activity level (ref: <2 times per week)		−0.004 (−0.040 to 0.032)	0.814	0.001	0.005 (−0.021 to 0.030)	0.725	0.002	0.010 (−0.017 to 0.038)	0.449	0.009
**Model summary**		R^2^ = 0.441	Adj. R^2^ = 0.381		R^2^ = 0.413	Adj. R^2^ = 0.351		R^2^ = 0.315	Adj. R^2^ = 0.242	

BMI—Body Mass Index; MEDAS-O—Mediterranean Diet Adherence Screener-Original; MEDAS-R—Mediterranean Diet Adherence Screener-Reverse scoring in wine; Ref.—Reference Category; Adj.—Adjusted; MoCA—Montreal Cognitive Assessment; ACE-R—Addenbrooke’s Cognitive Examination Revised; MMSE—Mini-Mental State Examination. Note: Adjusted for sex, age, BMI, education and physical activity level in 75 Portuguese adults at higher risk of dementia. Statistically significant results are shown in **bold** (*p* < 0.05).

## Data Availability

The raw data supporting the conclusions of this article will be made available by the authors upon request. The data are not publicly available because they consist of preliminary baseline data from an ongoing study.

## Supplementary Materials

The following supporting information can be downloaded at: https://www.mdpi.com/article/10.3390/nu17223576/s1, Table S1. Sex × MEDAS (both MEDAS-O and MEDAS-R) interaction analyses for each cognitive test.

## Abbreviations

The following abbreviations are used in this manuscript:

ACE-R	Addenbrooke’s Cognitive Examination Revised
ANCOVA	Analysis of Covariance
BMI	Body Mass Index
MD	Mediterranean Diet
MEDAS	Mediterranean Diet Adherence Screener
MEDAS-O	Mediterranean Diet Adherence Screener-Original
MEDAS-R	Mediterranean Diet Adherence Screener-Reverse Scoring in Wine
MMSE	Mini-Mental State Examination
MoCA	Montreal Cognitive Assessment
PCI	Probable Cognitive Impairment
SD	Standard Deviations
WHO	World Health Organisation
