# Supplementary material for: Challenging the Wine Component in Mediterranean Diet Scores: Cognitive Outcomes in Portuguese Adults at High Risk of Dementia
Source: Nutrients. 2025 Nov 15;17(22):3576. doi: 10.3390/nu17223576 (PMC12655420; doi:10.3390/nu17223576)
Supplement: Supplementary file 1 [file nutrients-17-03576-s001.zip › nutrients-3937315-supplementary.pdf]

Table S1. Sex × MEDAS (both MEDAS-O and MEDAS-R) interaction analyses for each cognitive test.

**Cognitive Tests**	***MEDAS-O***			***MEDAS-R***		
	Interaction Term	β (95% CI)	*p*-value	*Interaction Term*	β (95% CI)	*p*-value
**MoCA Score**	Sex*Medas-O	0.005 (-0.015 to 0.025)	0.590	Sex*Medas-R	0.012 (-0.009 to 0.034)	0.252
**ACE-R Score**	Sex*Medas-O	0.007 (-0.007 to 0.021)	0.341	Sex*Medas-R	0.014 (-0.001 to 0.029)	0.071
**MMSE Score**	Sex*Medas-O	0.008 (-0.007 to 0.023)	0.313	Sex*Medas-R	0.015 (-0.001 to 0.031)	0.074
